# Adjusted comparison of antegrade cerebral perfusion techniques during prolonged circulatory arrest in surgery for acute type A aortic dissection

**DOI:** 10.1016/j.xjon.2026.101817

**Published:** 2026-04-17

**Authors:** Leonard Pitts, Matteo Montagner, Markus Kofler, Gaik Nersesian, Julius Kaemmel, Semih Buz, Volkmar Falk, Jörg Kempfert

**Affiliations:** a Department of Cardiothoracic and Vascular Surgery, German Centre for Cardiovascular Research, Berlin, Germany; b Charité–Universitätsmedizin Berlin, Corporate Member of Freie Universität Berlin and Humboldt-Universität zu Berlin, Berlin, Germany; c German Center for Cardiovascular Research, Berlin, Germany; d Department of Health Sciences and Technology, Translational Cardiovascular Technologies, Institute of Translational Medicine, Swiss Federal Institute of Technology, Zurich, Switzerland

**Keywords:** acute type A aortic dissection, cerebral perfusion, malperfusion, stroke

## Abstract

**Objective:**

To investigate differences in neurologic outcomes between unilateral and bilateral antegrade cerebral perfusion during prolonged circulatory arrest in surgery for acute type A aortic dissection (ATAAD).

**Methods:**

Patients who underwent surgery for ATAAD between 2013 and 2023 receiving either unilateral antegrade cerebral perfusion (uACP) or bilateral antegrade cerebral perfusion (bACP) during prolonged caudal circulatory arrest (≥30 minutes) were included in the primary study cohort. Preoperative computed tomography scans were analyzed in terms of Type-Entry-Malperfusion classification and supra-aortic dissection patterns. After propensity score matching, the groups were compared in terms of clinical outcomes, including new postoperative strokes.

**Results:**

A total of 382 patients (206 uACP and 176 bACP) were included. The matched cohort comprised 2 balanced groups with 170 patients (85 in each group). The median circulatory arrest time was 44 (interquartile range [IQR], 36-57) minutes in the uACP group and 44 (IQR, 40-56) minutes in the bACP group (*P* = .87). Thirty-day mortality occurred in 15 patients (18%) in each group (odds ratio [OR], 1.00; 95% confidence interval [CI], 0.89-1.12; *P* = 1.00). New postoperative stroke was detected in 6 uACP patients (7%) and in 4 bACP patients (5%) (OR, 0.98; 95% CI, 0.91-1.05; *P* = .52). Postoperative delirium occurred in 30 uACP patients (35%) and in 35 bACP patients (41%) (OR, 1.06; 95% CI, 0.92-1.23; *P* = .43).

**Conclusions:**

Both uACP and bACP are adequate cerebral perfusion strategies during circulatory arrest exceeding 30 minutes in surgery for ATAAD. Additional intraoperative and anatomic factors may be considered to determine the optimal selective cerebral perfusion strategy.

## Graphical Abstract

**Figure:**
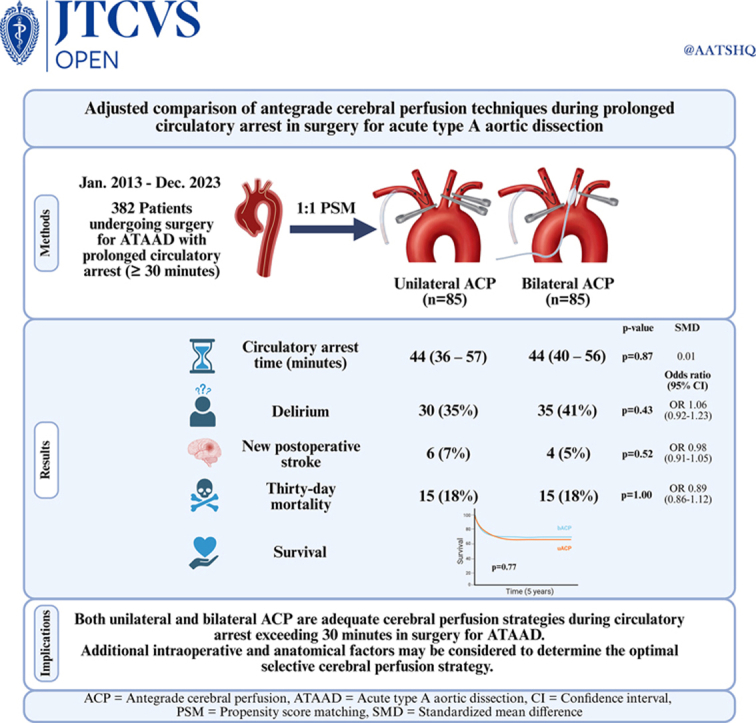

**Figure:**
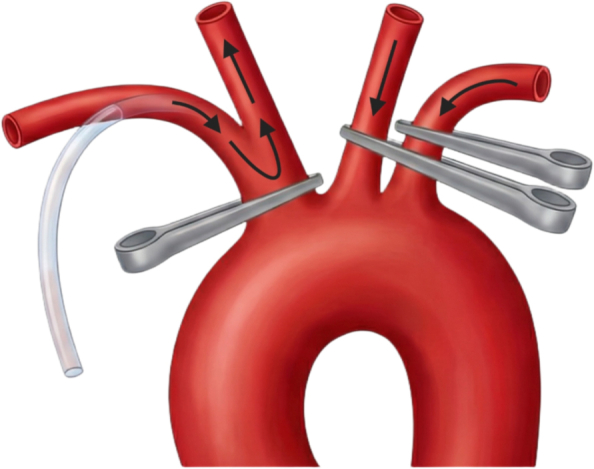
Unilateral ACP is simple and safe in surgery for ATAAD.

**Central Message**

Both uACP and bACP are adequate cerebral perfusion strategies during circulatory arrest exceeding 30 minutes.

**Perspective**

Due to its technical simplicity, uACP may serve as a valid cerebral perfusion strategy also in case of prolonged circulatory arrest for surgery in ATAAD. However, additional intraoperative and anatomical factors should be considered to determine the optimal selective cerebral perfusion strategy in each patient while adequate neuromonitoring is of utmost importance.

Acute type A aortic dissection (ATAAD) is associated with high mortality and requires urgent surgical repair.^1^ Intraoperative selective cerebral perfusion is of utmost importance to reduce the risk of perioperative stroke and restore cerebral blood flow in case of supra-aortic malperfusion.^2^^,^^3^ Current guidelines recommend antegrade cerebral perfusion (ACP) techniques, with a general consensus that deep hypothermic circulatory arrest alone should be avoided.^4^ The most frequently used techniques in Europe are unilateral ACP (uACP) and bilateral ACP (bACP) in combination with moderate hypothermia^5^; however, no clear recommendations exist for the use of either uACP or bACP. Although bACP has been proposed as the preferred technique in case of circulatory arrest times of more than 30 minutes, supporting evidence is highly limited.^6^ In this propensity score–matched study, we investigated differences in neurologic outcomes between patients undergoing surgery for ATAAD receiving uACP and those receiving bACP during prolonged circulatory arrest (≥30 minutes).

## Methods

### Ethics Approval

This study was granted Institutional Review Board approval (EA2/096/20, approved July 7, 2020) and was conducted in compliance with the Declaration of Helsinki. The need for active informed consent was waived owing to the study’s retrospective nature. The storage of participant data for multiple, indefinite research follows the standards set forth in the World Medical Association’s Declaration of Taipei.

### Patient Population and Preoperative Radiological Assessment

All patients with available preoperative computed tomography (CT) scans who underwent emergent surgery for ATAAD between 2013 and 2023 were included in the primary study cohort. Iatrogenic and subacute/chronic dissections (onset ≥14 days) were excluded. Patients undergoing deep hypothermic circulatory arrest with or without the use of retrograde cerebral perfusion were excluded.

Preoperative CT scans were analyzed according to dissection type (T), corresponding entry site (E), and radiological ± clinical signs of malperfusion (M±) following the recently published Type-Entry-Malperfusion classification.^7^ Additionally, the supra-aortic vessels (ie, innominate artery, right axillary artery, right common carotid artery, left common carotid artery, and left subclavian artery) were investigated for the extension of dissection. The definition of aortic arch anomalies included bovine aortic arch, isolated left vertebral artery, and arteria lusoria.

### Surgical Procedure and Selective Cerebral Perfusion

Cannulation of the right axillary artery was performed first, followed by median sternotomy, systemic heparinization, and venous cannulation for completion of cardiopulmonary bypass. The femoral artery, innominate artery, or aortic arch was cannulated only in selected scenarios. System cooling was initiated, aiming for moderate hypothermia at 28 °C (nasopharyngeal and bladder). Simultaneously, the innominate vein and the aortic arch, including the brachiocephalic trunk, left common carotid artery, and left subclavian artery, were surgically exposed. An arch-first approach was favored in most of the patients by reducing pump flow to 10 mL/kg/minute through the right axillary artery and clamping of the innominate artery for the initiation of uACP. After aortotomy, induction of cardioplegic arrest, and inspection of the entry site, the ascending aorta was resected either until zone 0/1 for hemiarch replacement or the aortic arch was resected until zone 2/3 for total arch replacement using the frozen elephant trunk (FET) technique, both in combination with an open distal anastomosis. FET implantation also was performed in the event of an aneurysm or entry tear located in the arch or descending aorta. Based on the preoperative CT scan and according to the surgeon's decision, additional stent (Ascyrus Medical Dissection Stent [AMDS]; Artivion) implantation to ascending/hemiarch replacement was performed since 2018 in selected patients.^8^

In cases of uACP, the left common carotid artery was occluded. For expansion to bACP, additional direct cannulation of the left common carotid artery using a ballon-tipped catheter was performed. To prevent the steal phenomenon, the left subclavian artery was occluded in most cases. Intraoperative neuromonitoring through near-infrared spectroscopy (NIRS) was not available for several patients, given that its use began in 2013 and was consequently performed since 2016. The choice of uACP or bACP was mainly surgeon-dependent. An intraoperative switch from uACP to bACP was performed only in cases with asymmetric NIRS values (a significant NIRS drop over the left-sided hemisphere ≈15%) and/or reduced arterial backflow of the left common carotid artery.

The proximal anastomosis was made in accordance with aortic root surgery; if possible, the aortic root was reconstructed using felt sutures and glue in a “sandwich” technique, and the aortic valve was reconstructed by resuspension of the commissures. Valve-sparing root replacement was restricted to isolated cases and performed primarily in young and hemodynamically stable patients. In all other cases, composite aortic valve and root replacement with a valved conduit and reimplantation of the coronary arteries were performed.

### Outcomes and Definition of Stroke

The primary endpoint was new postoperative stroke (ischemic and/or hemorrhagic stroke and/or cerebral edema) confirmed by cerebral CT scan. “New” was defined by the absence of preoperative radiological and/or clinical cerebral malperfusion (not M2+/−) while the time point of diagnosis had to be “operation-related” (ie, diagnosis was made immediately after narcosis termination at the first postoperative neurologic examination). In contrast, delayed stroke was defined as any CT-confirmed stroke occurring during further treatment and not operation-related (eg, due to postoperative reanimation, reintervention, mechanical circulatory support, etc). Secondary endpoints included postoperative delirium, 30-day mortality. and survival. Postoperative delirium was performed by clinical assessment on the intensive care unit and the ward during the postoperative course.

The follow-up was performed by telephone calls and postoperative appointments in our aortic department. The median duration of follow-up was 1264 days, and the study was closed in June 2025. Follow-up did not differ between the 2 treatment groups (median, 1256 days vs 1272 days; *P* = .66).

### Statistical Analysis

**Figure 1:**
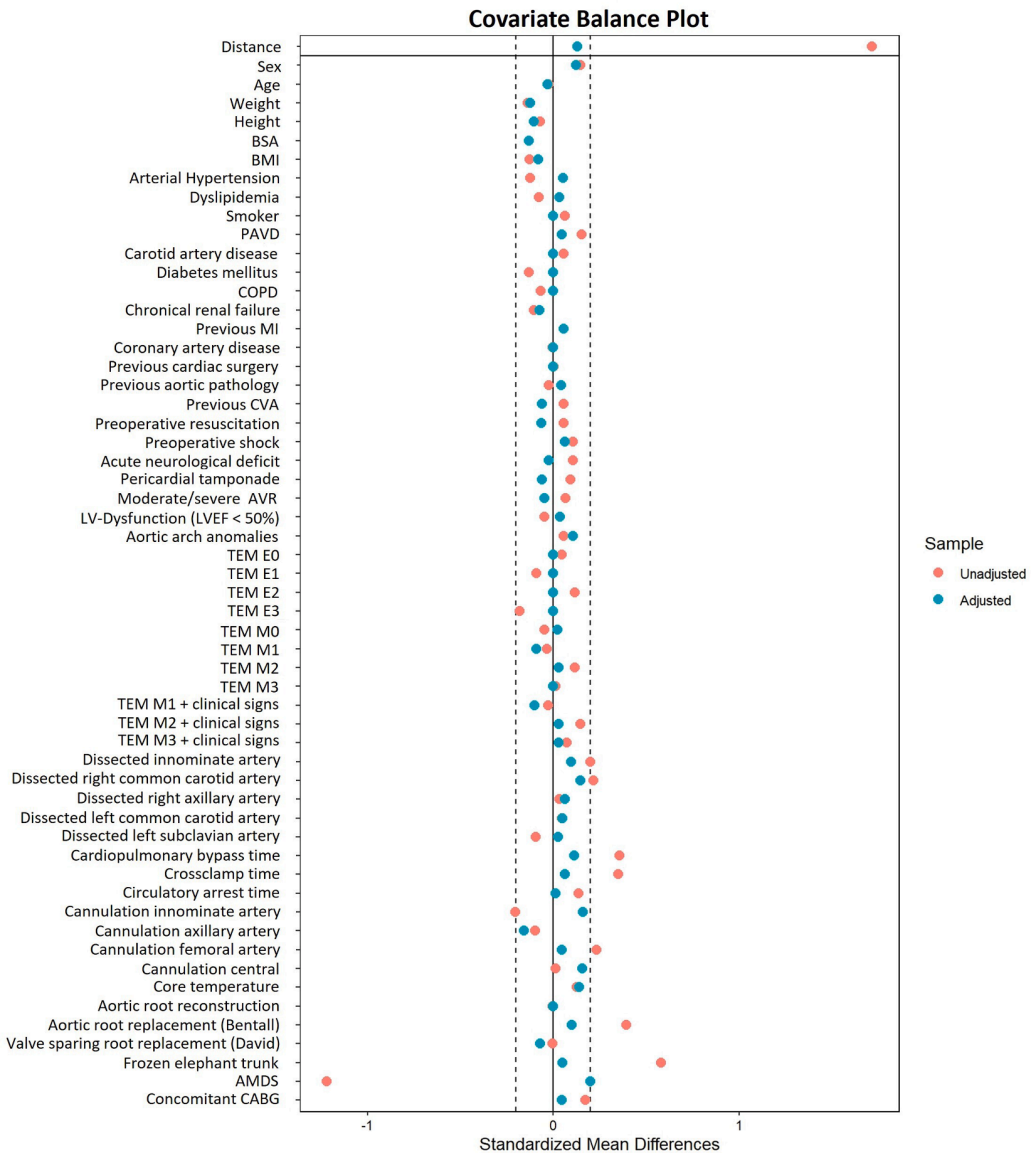
Covariate balance plot. *BSA*, Body surface area; *BMI*, body mass index; *PAVD*, peripheral arterial vascular disease; *COPD*, chronic obstructive pulmonary disease; *MI*, myocardial infarction; *CVA*, cerebrovascular accident; *AVR*, aortic valve regurgitation; *LV*, left ventricle; *LVEF*, left ventricular ejection fraction; *TEM*, Type-Entry-Malperfusion; *AMDS*, Ascyrus Medical Dissection Stent; *CABG*, coronary artery bypass grafting.

Continuous variables were tested for normal distribution (Shapiro-Wilk test combined with visualization using histograms). The variables non-normally distributed and thus are presented as median with corresponding interquartile range (IQR, 25th-75th percentile) and compared using the Wilcoxon rank-sum test. The paired Wilcoxon rank-sum test was used for matched data. Categorical data are represented as absolute numbers with corresponding percentages and compared using the χ^2^ test or Fisher exact test in case of low frequency, while the McNemar test was used for matched data. Propensity score matching (1:1) via the nearest-neighbor method using a caliper width of 0.2 with the R “MatchItP” package, version 4.5.5 (R Foundation for Statistical Computing), considering all clinical preoperative variables (Table 1), preoperative CT-based variables (Table 2), and intraoperative variables (Table 3) was performed to balance the groups regarding important preoperative and intraoperative variables as well as potential confounders. The covariate balance plot in Figure 1 illustrates the corresponding standardized mean differences, which were defined as acceptable until ±0.20. The association between treatment (ie, bACP) and outcome was measured using logistic regression analysis generating odds ratio (OR) with 95% confidence interval (CI). A Kaplan-Meier curve including log-rank test was designed to illustrate and compare survival between both groups over a 5-year period. All *P* values are 2-sided. The α-level was defined at 0.05. Statistical analysis was performed using R version 4.3.2.

**Table 1:** Preoperative variables (postmatch)

Variable	Total (N = 170)	uACP (N = 85)	bACP (N = 85)	*P* value (α = .05)	SMD
Female sex, n (%))	61 (36)	28 (33)	33 (39)	.70	0.12
Age, y, median (IQR)	65 (55-74)	65 (54-75)	64 (56-73)	.86	−0.03
Weight, kg, median (IQR)	80 (70-92)	80 (70-95)	80 (70-90)	.28	−0.12
Height, cm, median (IQR)	176 (168-182)	175 (168-183)	176 (168-182)	.38	−0.10
BSA, m^2^, median (IQR)	1.98 (1.81-2.15)	2.00 (1.83-2.16)	1.97 (1.81-2.13)	.21	−0.13
BMI, kg/m^2^, median (IQR)	26.2 (23.6-28.9)	26.3 (23.7-29.4)	26.2 (23.4-28.4)	.48	−0.08
Arterial hypertension, n (%)	132 (78)	65 (76)	67 (79)	.87	0.05
Dyslipidemia, n (%)	31 (18)	15 (18)	16 (19)	1.00	0.03
Smoker, n (%)	58 (34)	29 (34)	29 (34)	.93	0.00
PAVD, n (%)	7 (4)	3 (4)	4 (5)	1.00	0.04
Carotid artery disease, n (%)	4 (2)	2 (2)	2 (2)	.94	0.00
Diabetes mellitus, n (%)	8 (5)	4 (5)	4 (5)	.94	0.00
COPD, n (%)	10 (6)	5 (6)	5 (6)	.94	0.00
Chronic renal failure, n (%)	20 (12)	11 (13)	9 (11)	.93	−0.07
Coronary artery disease, n (%)	22 (13)	11 (13)	11 (13)	.93	0.00
Previous MI, n (%)	11 (6)	5 (6)	6 (7)	1.00	0.05
Previous CVA, n (%)	7 (4)	4 (5)	3 (4)	1.00	−0.06
Previous cardiac surgery, n (%)	8 (5)	4 (5)	4 (5)	.94	0.00
Previous aortic pathology, n (%)	19 (11)	9 (11)	10 (12)	1.00	0.04
LV dysfunction (LVEF <50%), n (%)	27 (16)	13 (15)	14 (16)	1.00	0.03
Moderate/severe AVR, n (%)	64 (38)	33 (39)	31 (36)	.92	−0.04
Pericardial tamponade, n (%)	30 (18)	16 (19)	14 (16)	.93	−0.06
Acute neurologic deficit, n (%)	49 (29)	25 (29)	24 (28)	1.00	−0.02
Preoperative shock, n (%)	30 (18)	14 (16)	16 (19)	.93	0.06
Preoperative resuscitation, n (%)	5 (3)	3 (4)	2 (2)	1.00	−0.06

*uACP*, Unilateral antegrade cerebral perfusion; *bACP*, bilateral antegrade cerebral perfusion; *SMD*, standardized mean difference; *IQR*, interquartile range; *BMI*, body mass index; *BSA*, body surface area; *PAVD*, peripheral arterial vascular disease; *COPD*, chronic obstructive pulmonary disease; *MI*, myocardial infarction; *CVA*, cerebrovascular accident; *LVEF*, left ventricular ejection fraction; *AVR*, aortic valve regurgitation.

**Table 2:** Preoperative CT-based variables (postmatch)

Variable	Total (N = 170)	uACP (N = 85)	bACP (N = 85)	*P* value (α = .05)	SMD
Entry site, n (%)					
E0 (nondetectable)	14 (8)	7 (8)	7 (8)	.94	0.00
E1 (ascending aorta)	138 (81)	69 (81)	69 (81)	.86	0.00
E2 (aortic arch)	18 (11)	9 (11)	9 (11)	.94	0.00
E3 (descending aorta)	0 (0)	0 (0)	0 (0)	1.00	0.00
Malperfusion, n (%)					
M0 (no malperfusion)	109 (64)	54 (64)	55 (65)	1.00	0.02
M1 (coronary malperfusion)	12 (7)	7 (8)	5 (6)	.94	−0.09
Clinical signs	6 (4)	4 (5)	2 (2)	.94	−0.10
M2 (supra-aortic malperfusion)	35 (21)	17 (20)	18 (21)	1.00	0.02
Clinical signs	27 (16)	13 (15)	14 (16)	1.00	0.02
M3 (spinal, visceral, iliac)	32 (19)	16 (19)	16 (19)	.93	0.00
Clinical signs	25 (15)	12 (14)	13 (15)	1.00	0.02
Aortic arch anomalies, n (%)	29 (17)	13 (15)	16 (19)	.86	0.10
Supra-aortic vessel involvement, n (%)					
Innominate artery	94 (55)	45 (53)	49 (58)	.73	0.09
Right common carotid artery	54 (32)	24 (28)	30 (35)	.64	0.14
Right axillary artery	26 (15)	12 (14)	14 (16)	.93	0.06
Left common carotid artery	52 (31)	25 (29)	27 (32)	.93	0.04
Left subclavian artery	65 (38)	32 (38)	33 (39)	1.00	0.02

*CT*, Computed tomography; *uACP*, unilateral antegrade cerebral perfusion; *bACP*, bilateral antegrade cerebral perfusion; *SMD*, standardized mean difference.

**Table 3:** Intraoperative variables (postmatch)

Variable	Total (N = 170)	uACP (N = 85)	bACP (N = 85)	*P* value (α = .05)	SMD
CPB time, min, median (IQR)	219 (177-264)	221 (175-260)	217 (182-275)	.54	0.11
Cross-clamp time, min, median (IQR)	105 (88-130)	105 (86-128)	105 (88-133)	.81	0.06
Circulatory arrest time, min, median (IQR)	44 (38-56)	44 (36-57)	44 (40-56)	.87	0.01
Arterial cannulation, n (%)					
Innominate artery	6 (4)	2 (2)	4 (5)	.94	0.15
Right axillary artery	158 (93)	81 (95)	77 (91)	.39	−0.15
Right femoral artery	5 (3)	2 (2)	3 (4)	1.00	0.04
Central	1 (1)	0 (0)	1 (1)	.94	0.15
Core temperature, °C, median (IQR)	28 (26-28)	28 (25-28)	28 (26-28)	.50	0.13
Aortic root reconstruction	90 (53)	45 (53)	45 (53)	.91	0.00
Aortic root replacement (Bentall)	42 (25)	19 (22)	23 (27)	.79	0.09
Valve-sparing root replacement (David)	3 (2)	2 (2)	1 (1)	1.00	−0.07
AMDS	23 (14)	9 (11)	14 (16)	.74	0.19
Frozen elephant trunk	22 (13)	10 (12)	12 (14)	.93	0.05
Concomitant CABG	7 (4)	3 (4)	4 (5)	1.00	0.04

*uACP*, Unilateral antegrade cerebral perfusion; *bACP*, bilateral antegrade cerebral perfusion; *SMD*, standardized mean difference; *CPB*, cardiopulmonary bypass; *AMDS*, Ascyrus Medical Dissection Stent; *CABG*, coronary artery bypass grafting.

## Results

### Preoperative Variables

**Figure 2:**
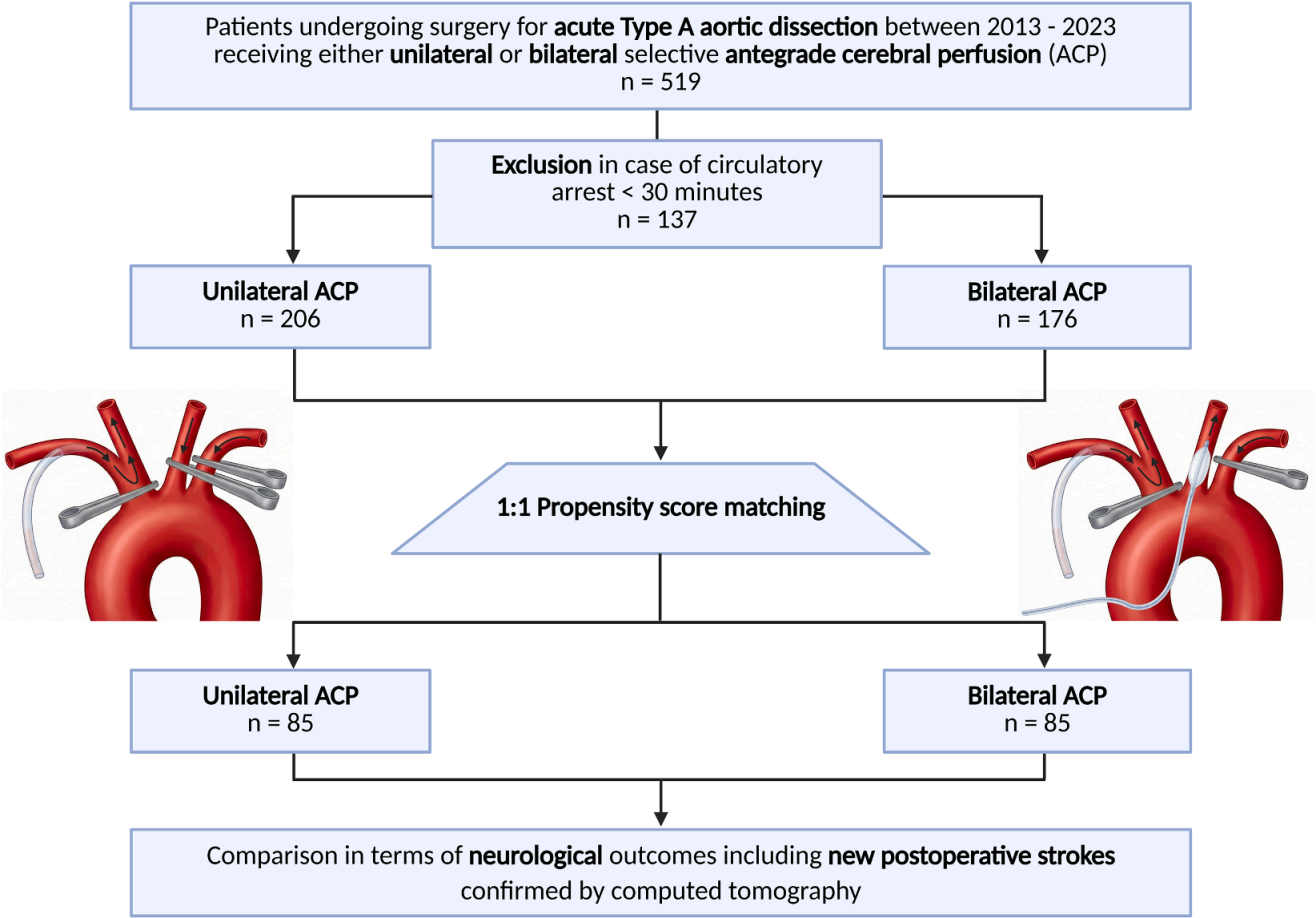
Study flowchart. *ACP*, Antegrade cerebral perfusion.

After exclusion of patients who received no selective cerebral perfusion (n = 54) or retrograde cerebral perfusion (n = 76), as well as those with a circulatory arrest time <30 minutes (n = 137), the final study cohort included 382 patients, with 206 patients in the uACP group and 176 in the bACP group. After 1:1 matching, the final study cohort comprised 170 patients (85 matched pairs). A corresponding study flowchart for patient selection and group formation is shown in Figure 2. The preoperative variables, including the corresponding CT-based variables, for the matched cohorts are shown in Tables 1 and 2. After matching, all variables demonstrated well-balanced results between the 2 treatment groups (for unmatched data are provided in Tables E1 and E2). Cerebral malperfusion was present in 17 uACP patients (20%) and 18 bACP patients (21%) (*P* = 1.00), whereas 13 (15%) and 14 (16%), respectively, also showed clinical signs (*P* = 1.00). The innominate artery was dissected in 45 uACP patients (53%) and 49 bACP patients (58%) (*P* = .73), followed by the left subclavian artery in 32 (38%) versus 33 (39%) (*P* = 1.00), the right common carotid artery in 24 (28%) versus 30 (35%) (*P* = .64), the left common carotid artery in 25 (29%) versus 27 (32%) (*P* = .93), and the right axillary artery in 12 (14%) versus 14 (16%) (*P* = .93).

### Intraoperative Variables

The intraoperative variables for the matched cohorts are shown in Table 3 (for unmatched data, see Table E3) and demonstrate well balanced results between both treatment groups. Most patients underwent ascending/hemiarch replacement in combination with an open distal anastomosis in zone 0 or—in a few patients—zone 1, whereas additional AMDS implantation in these patients was performed in 9 patients (11%) in the uACP group and 14 patients (16%) in the bACP group (*P* = .74). In comparison, total arch replacement using the FET was performed in 10 patients (12%) in the uACP group and 12 patients (14%) in the bACP group in zone 2/3 (*P* = .93). Primary cannulation of the femoral artery was performed in only 5 patients (2 in the uACP group and 3 in the bACP group). The decision for femoral cannulation was made on a case-by-case decision and did not represent the standard cannulation technique. In these patients, the cannulation technique for ACP differed in every patient and depended on the individual situation and surgeon preference; details are summarized in Table E5).

The median circulatory arrest time was 44 (IQR, 36-57) minutes in the uACP group and 44 (IQR, 40-56) minutes in the bACP group (*P* = .87). Two patients undergoing uACP experienced a significant left-sided NIRS drop and reduced backflow of the left common carotid artery, resulting in expansion to bACP soon after initialization of selective cerebral perfusion.

### Outcomes

**Figure 3:**
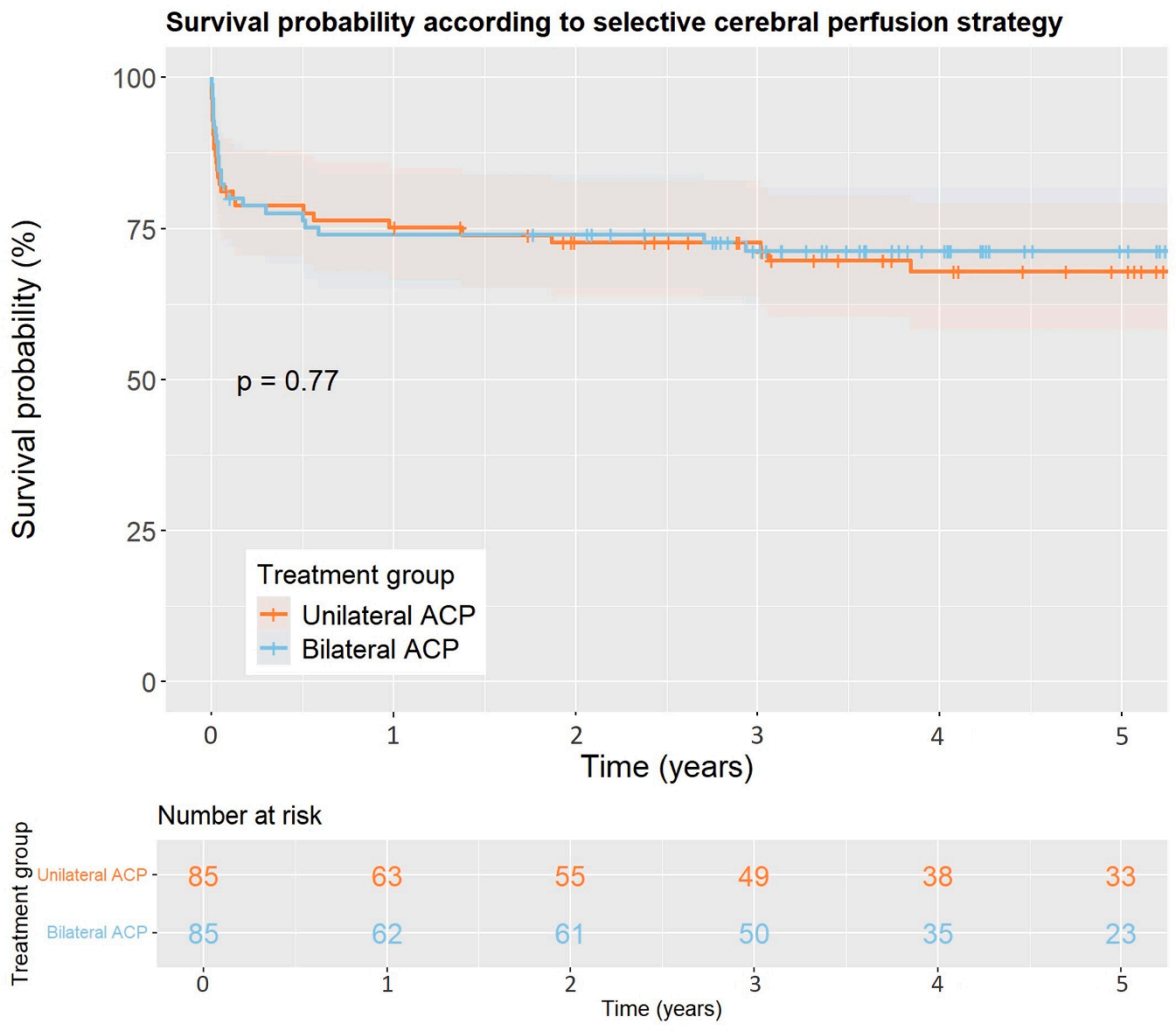
Survival according to treatment group, with 95% confidence intervals. *ACP*, Antegrade cerebral perfusion.

Postoperative variables for the matched cohorts are shown in Table 4 (for unmatched data, see Table E4). Thirty-day mortality occurred in 15 patients (18%) in each group (OR, 1.00; 95% CI, 0.89-1.12; *P* = 1.00). New postoperative stroke was diagnosed in 6 patients (7%) of the uACP group and 4 patients (5%) of the bACP group (OR, 0.98; 95% CI, 0.91-1.05; *P* = .52). Delayed stroke not related to the index surgery occurred in 6 patients (7%) of each group (OR, 1.00; 95% CI, 0.93-1.07; *P* = 1.00). Postoperative delirium was detected in 30 patients (35%) of the uACP group and 35 (41%) patients of the bACP group (OR, 1.06; 95% CI, 0.92-1.23; *P* = .43). Further outcomes did not differ between the 2 groups. Five-year survival, illustrated as a Kaplan-Meier curve and including a log-rank test for comparison in Figure 3, did not differ significantly between the matched cohorts (*P* = .77).

**Table 4:** Postoperative variables (postmatch)

Variable	Total (N = 170)	uACP (N = 85)	bACP (N = 85)	*P* value (α = .05)	OR (95% CI)
ICU treatment time, d, median (IQR)	8 (3-18)	8 (3-18)	7 (3-18)	.75	1.93 (0.04-101.28)
Ventilation time, d, median (IQR)	3 (1-12)	3 (1-12)	3 (1-12)	.57	3.09 (0.06-154.27)
Reintubation	19 (11)	12 (14)	7 (8)	.23	0.94 (0.86-1.04)
Tracheotomy	32 (19)	18 (21)	14 (16)	.44	0.95 (0.85-1.07)
Open chest treatment	28 (16)	18 (21)	10 (12)	.099	0.91 (0.81-1.02)
Postoperative ECLS	13 (8)	7 (8)	6 (7)	.77	0.99 (0.91-1.07)
Postoperative CRRT	28 (18)	14 (18)	14 (18)	.97	0.99 (0.89-1.12)
Revision for bleeding	43 (25)	17 (20)	26 (31)	.114	1.11 (0.98-1.27)
Revision for malperfusion	15 (9)	7 (8)	8 (9)	.79	1.01 (0.93-1.10)
Delirium	65 (38)	30 (35)	35 (41)	.43	1.06 (0.92-1.23)
New postoperative stroke	10 (6)	6 (7)	4 (5)	.52	0.98 (0.91-1.05)
Delayed stroke	12 (7%)	6 (7%)	6 (7%)	1.00	1.00 (0.93-1.07)
New spinal ischemia	2 (1)	2 (2)	0 (0)	.157	0.98 (0.95-1.01)
Thirty-day mortality	30 (18)	15 (18)	15 (18)	1.00	1.00 (0.89-1.12)

*uACP*, Unilateral antegrade cerebral perfusion; *bACP*, bilateral antegrade cerebral perfusion; *OR*, odds ratio; *CI*, confidence interval; *ICU*, intensive care unit; *ECLS*, extracorporeal life support, *CRRT*, continuous renal replacement therapy.

## Discussion

This is the first propensity score–matched study investigating the neurologic outcomes between uACP and bACP during prolonged circulatory arrest in patients undergoing surgery for ATAAD. The present results suggest that similar outcomes may be achieved by each technique under certain circumstances in the event of distal arrest times ≥30 minutes, while the median circulatory arrest time was 45 minutes in both groups. To elaborate on the true effect of selective cerebral perfusion techniques in ATAAD, we believe that propensity score matching is of utmost importance, considering clinical and radiological data for cerebral malperfusion, including supra-aortic dissection patterns, as well as the corresponding circulatory arrest time. Several previous studies have compared uACP to bACP in the setting of ATAAD, but most could not demonstrate a clear superiority of one technique over the other.^6^^,^^9^^,^^10^ According to this, a recent meta-analysis showed no differences in terms of neurologic outcomes between the 2 techniques in surgery for ATAAD.^11^ A subgroup analysis by Angleitner and colleagues^12^ demonstrated improved long-term survival with bACP compared to uACP when circulatory arrest was maintained for ≥50 minutes. Liu and colleagues^13^ reported a significantly reduced risk for stroke with bACP compared to uACP in a large series of 321 ATAAD patients. However, those studies either were not adjusted for important confounders or lacked important data in terms of cerebral malperfusion and supra-aortic dissection patterns.

A possible argument raised against the use of bACP is the additional manipulation of supra-aortic vessels; however, this remains speculative and currently is not supported by any reliable data. Studies promoting the use of uACP because of its simplicity often include shorter circulatory arrest times (<30 minutes) and more hemiarch replacements instead of complex arch surgery, while bACP is the preferred technique for estimated prolonged distal circulatory arrest.^10^^,^^14^ The present study aimed to eliminate this bias, highlighting that the estimated circulatory arrest time alone might not be the sole driving factor if precise neuromonitoring is provided and individual anatomic and technical aspects are adequately considered. The use of uACP assumes the presence of an intact circle of Willis, which either is not observed consistently in patients undergoing surgery for ATAAD or often is not provided by preoperative CT scans.^15^ However, the anatomic integrity of the circle of Willis does not necessarily correlate with its functionality.^16^ Although there are no available data as yet, it might be advantageous to use trilateral ACP in selected cases by additional cannulation of the left subclavian artery in patients with an incomplete circle of Willis.

### Limitations

This study is limited by its retrospective and unicentric nature, which may significantly impact the generalizability of the results. Intraoperative NIRS was not available for all patients between 2013 and 2016, which may carry the risk of bias and influence results, although the incidence of stroke was distributed equally over the study period.

## Conclusions

This study provides an additional puzzle piece in the debate about cerebral perfusion strategies for surgical treatment of ATAAD. Because of its technical simplicity, uACP may be a valid cerebral perfusion strategy also in cases of prolonged circulatory arrest for surgery in ATAAD. While adequate intraoperative neuromonitoring plays an important role, additional intraoperative and anatomic factors should be taken into account to determine the optimal cerebral perfusion technique for each patient.

## Conflict of Interest Statement

The authors reported no conflicts of interest.

The *Journal* policy requires editors and reviewers to disclose conflicts of interest and to decline handling or reviewing manuscripts for which they may have a conflict of interest. The editors and reviewers of this article have no conflicts of interest.

## Figures and Tables

**Table E1:** Preoperative variables (prematch)

Variable	Total (N = 382)	uACP (N = 206)	bACP (N = 176)	*P* value (α = .05)	SMD
Female sex, n (%)	131 (34)	64 (31)	67 (38)	.152	0.14
Age, y, median (IQR)	63 (54-74)	62 (54-73)	63 (54-75)	.88	−0.02
Weight, kg, median (IQR)	80 (70-91)	81 (72-92)	80 (70-90)	.22	−0.14
Height, cm, median (IQR)	175 (168-182)	175 (168-181)	175 (167-182)	.57	−0.07
BSA, m^2^, median (IQR)	2.00 (1.83-2.14)	2.00 (1.84-2.15)	1.97 (1.82-2.12)	.27	−0.13
BMI, kg/m^2^, median (IQR)	26.2 (23.9-28.7)	26.3 (24.1-29.4)	25.9 (23.9-27.9)	.106	−0.13
Arterial hypertension, n (%)	283 (74)	158 (77)	125 (71)	.21	−0.13
Dyslipidemia, n (%)	69 (18)	40 (19)	29 (16)	.46	−0.08
Smoker, n (%)	135 (35)	70 (34)	65 (37)	.55	0.06
PAVD, n (%)	20 (5)	7 (3)	13 (7)	.081	0.15
Carotid artery disease, n (%)	7 (2)	3 (1)	4 (2)	.55	0.05
Diabetes mellitus, n (%)	23 (6)	15 (7)	8 (5)	.26	−0.13
COPD, n (%)	25 (7)	15 (7)	10 (6)	.53	−0.07
Chronical renal failure, n (%)	48 (13)	29 (14)	19 (11)	.34	−0.11
Coronary artery disease, n (%)	35 (9)	19 (9)	16 (9)	.96	−0.01
Previous MI, n (%)	15 (4)	7 (3)	8 (5)	.57	0.06
Previous CVA, n (%)	13 (3)	6 (3)	7 (4)	.57	0.05
Previous cardiac surgery, n (%)	13 (3)	7 (3)	6 (3)	.99	0.00
Previous aortic pathology, n (%)	34 (9)	19 (9)	15 (9)	.81	−0.03
LV dysfunction (LVEF <50%), n (%)	51 (13)	29 (14)	22 (12)	.65	−0.05
Moderate/severe AVR, n (%)	154 (40)	80 (39)	74 (42)	.53	0.07
Pericardial tamponade, n (%)	60 (16)	29 (14)	31 (18)	.35	0.09
Acute neurologic deficit, n (%)	120 (31)	60 (29)	60 (34)	.30	0.10
Preoperative shock, n (%)	61 (16)	29 (14)	32 (18)	.28	0.11
Preoperative resuscitation, n (%)	11 (3)	5 (2)	6 (3)	.57	0.05

*uACP*, Unilateral antegrade cerebral perfusion; *bACP*, bilateral antegrade cerebral perfusion; *SMD*, standardized mean difference; *IQR*, interquartile range; *BSA*, body surface area; *BMI*, body mass index; *PAVD*, peripheral arterial vascular disease; *COPD*, chronic obstructive pulmonary disease; *MI*, myocardial infarction; *CVA*, cerebrovascular accident; *LVEF*, left ventricular ejection fraction; *AVR*, aortic valve regurgitation.

**Table E2:** Preoperative CT-based variables (prematch)

Variable	Total (N = 382)	uACP (N = 206)	bACP (N = 176)	*P* value (α = .05)	SMD
Entry site, n (%)					
E0 (nondetectable)	32 (8)	16 (8)	16 (9)	.64	0.05
E1 (ascending aorta)	303 (79)	167 (81)	136 (77)	.36	−0.09
E2 (aortic arch)	42 (11)	19 (9)	23 (13)	.23	0.11
E3 (descending aorta)	5 (1)	4 (2)	1 (1)	.24	−0.18
Malperfusion, n (%)					
M0 (no malperfusion)	224 (59)	123 (60)	101 (57)	.65	−0.05
M1 (coronary malperfusion)	30 (8)	17 (8)	13 (7)	.76	−0.03
Clinical signs	23 (6)	13 (6)	10 (6)	.80	−0.03
M2 (supra-aortic malperfusion)	83 (22)	40 (19)	43 (24)	.24	0.12
Clinical signs	64 (17)	29 (14)	35 (20)	.130	0.15
M3 (spinal, visceral, iliac)	90 (24)	48 (23)	42 (24)	.90	0.01
Clinical signs	70 (18)	35 (17)	35 (20)	.47	0.07
Aortic arch anomalies, n (%)	44 (12)	22 (11)	22 (12)	.58	0.06
Supra-aortic vessel involvement, n (%)					
Innominate artery	210 (55)	104 (50)	106 (60)	.057	0.20
Right common carotid artery	124 (32)	57 (28)	67 (38)	.031	0.21
Right axillary artery	67 (18)	35 (17)	32 (18)	.76	0.03
Left common carotid artery	128 (34)	67 (33)	61 (35)	.66	0.05
Left subclavian artery	146 (38)	83 (40)	63 (36)	.37	−0.09

*CT*, Computed tomography; *uACP*, unilateral antegrade cerebral perfusion; *bACP*, bilateral antegrade cerebral perfusion; *SMD*, standardized mean difference.

**Table E3:** Intraoperative variables (prematch)

Variable	Total (N = 382)	uACP (N = 206)	bACP (N = 176)	*P* value (α = .05)	SMD
CPB time, min, median (IQR)	213 (177-266)	204 (171-241)	222 (189-286)	<.001	0.36
Cross-clamp time, min, median (IQR)	106 (88-135)	100 (84-125)	116 (94-140)	<.001	0.35
Circulatory arrest time, min, median (IQR)	44 (37-55)	43 (36-53)	44 (38-57)	.172	0.14
Arterial cannulation, n (%)					
Innominate artery	15 (4)	11 (4)	4 (2)	.125	−0.21
Right axillary artery	351 (92)	192 (94)	159 (90)	.31	−0.10
Right femoral artery	14 (4)	2 (1)	12 (7)	.002	0.23
Central	2 (1)	1 (1)	1 (1)	.91	0.01
Core temperature, °C, median (IQR)	28 (26-28)	28 (26-28)	28 (26-28)	.89	0.12
Aortic root reconstruction, n (%)	187 (49)	101 (49)	86 (49)	.97	−0.01
Aortic root replacement (Bentall), n (%)	102 (27)	37 (18)	65 (37)	<.001	0.39
Valve-sparing root replacement (David), n (%)	11 (3)	6 (3)	5 (3)	.97	−0.01
AMDS, n (%)	111 (29)	94 (46)	17 (10)	<.001	−1.22
Frozen elephant trunk, n (%)	66 (17)	10 (5)	56 (32)	<.001	0.58
Concomitant CABG, n (%)	19 (5)	6 (3)	13 (7)	.045	0.17

*uACP*, Unilateral antegrade cerebral perfusion; *bACP*, bilateral antegrade cerebral perfusion; *SMD*, standardized mean difference; *CPB*, cardiopulmonary bypass; *AMDS*, Ascyrus Medical Dissection Stent; *CABG*, coronary artery bypass grafting.

**Table E4:** Postoperative variables (prematch)

Variable	Total (N = 382)	uACP (N = 206)	bACP (N = 176)	*P* value (α = .05)	OR (95% CI)
ICU treatment time, d, median (IQR)	8 (3-17)	8 (3-17)	7 (3-17)	.95	0.90 (0.03-24.80)
Ventilation time, d, median (IQR)	3 (1-12)	3 (1-10)	3 (1-13)	.35	4.03 (0.22-75.40)
Reintubation, n (%)	48 (13)	27 (13)	21 (12)	.73	0.99 (0.92-1.06)
Tracheotomy, n (%)	73 (19)	38 (18)	35 (20)	.72	1.01 (0.94-1.10)
Open chest treatment, n (%)	49 (13)	31 (15)	18 (10)	.161	0.95 (0.89-1.02)
Postoperative ECLS, n (%)	24 (6)	15 (7)	9 (5)	.36	0.98 (0.93-1.03)
Postoperative CRRT, n (%)	62 (17)	30 (16)	32 (19)	.37	1.04 (0.96-1.12)
Revision for bleeding, n (%)	82 (21)	42 (20)	40 (23)	.58	1.02 (0.94-1.11)
Revision for malperfusion, n (%)	52 (14)	27 (13)	25 (14)	.76	1.01 (0.94-1.08)
Delirium, n (%)	154 (40)	83 (40)	71 (40)	.99	1.00 (0.91-1.10)
New postoperative stroke, n (%)	34 (9)	16 (8)	18 (10)	.40	1.02 (0.97-1.09)
New spinal ischemia, n (%)	5 (1)	2 (1)	3 (2)	.53	1.01 (0.98-1.03)
Thirty-day mortality, n (%)	71 (19)	34 (17)	37 (21)	.26	1.05 (0.97-1.13)

*uACP*, Unilateral antegrade cerebral perfusion; *bACP*, bilateral antegrade cerebral perfusion; *OR*, odds ratio; *CI*, confidence interval; *ICU*, intensive care unit; *ECLS*, extracorporeal life support; *CRRT*, continuous renal replacement therapy.

**Table E5:** Cannulation and perfusion strategies in patients with primary femoral artery cannulation

#1 uACP	Femoral cannulation due to obliteration of the right subclavian artery, true lumen collapse of the downstream aorta and malperfusion of the right kidney. Initialization of cardiopulmonary bypass to expand true lumen flow followed by open cannulation of the right axillary artery under direct view for uACP.
#2 uACP	Peripheral malperfusion of lower extremities as well as coronary malperfusion due to coronary dissection of the right coronary artery (Neri C). Initial femoral cannulation to maintain circulation and expand the true lumen, followed by cannulation of the right axillary artery for uACP.
#1 bACP	Femoral cannulation due to obliteration of the innominate artery and extensive dissection of the supra-aortic vessels. For bACP, both carotid arteries were selectively cannulated and debranched followed by total arch replacement using the frozen elephant trunk technique. The right subclavian artery was also debranched in extra-anatomic fashion because the innominate artery was not repairable.
#2 bACP	Severe dissection and obliteration of the right common carotid artery with consecutive left sided hemiparesis. Femoral artery cannulation for initialization of cardiopulmonary bypass and open preparation of the right common carotid artery, followed by end-to-end anastomosis of a Dacron graft and start of selective uACP with an additional pump. After opening of the aortic arch, additional cannulation of the left common carotid artery for bACP. Extra-anatomic bypass of the right common carotid artery was performed as last step of the procedure.
#3 bACP	Severe dissection and obliteration of the right common carotid artery with diffuse neurologic symptoms. Femoral artery cannulation for initialization of cardiopulmonary bypass and open preparation of the right common carotid artery followed by end-to-end anastomosis of a Dacron graft and start of selective uACP with an additional pump. After opening of the aortic arch, additional cannulation of the left common carotid artery for bACP. Extra-anatomic bypass of the right common carotid artery was performed as the final step of the procedure.

*uACP*, Unilateral antegrade cerebral perfusion; *bACP*, bilateral antegrade cerebral perfusion.
